# The effectiveness of the *Inspiring Futures* parenting programme in improving behavioural and emotional outcomes in primary school children with behavioural or emotional difficulties: study protocol for a randomised controlled trial

**DOI:** 10.1186/s40359-018-0214-7

**Published:** 2018-02-20

**Authors:** Nick Axford, Georgina Warner, Tim Hobbs, Sarah Heilmann, Anam Raja, Vashti Berry, Obioha C. Ukoumunne, Justin Matthews, Tim Eames, Angeliki Kallitsoglou, Sarah Blower, Tom Wilkinson, Luke Timmons, Gretchen Bjornstad

**Affiliations:** 1 NIHR CLAHRC South West Peninsula (PenCLAHRC), Plymouth University Peninsula Schools of Medicine and Dentistry, ITTC, Plymouth Science Park, Plymouth, PL6 8BX UK; 2 Autistica, St Saviour’s House, 39-41 Union Street, London, SE1 1SD UK; 3 Dartington Service Design Lab, Lower Hood Barn, Dartington, TQ9 6AB UK; 4 Department of Public Administration and Sociology, Erasmus School of Social and Behavioural Sciences, Erasmus University Rotterdam, Mandeville Building, Burgemeester Oudlaan 50, 3062 PA, Rotterdam, T16-37 The Netherlands; 5 Department of Psychiatry, University of Oxford, Warneford Hospital, Oxford, OX3 7JX UK; 6 NIHR CLAHRC South West Peninsula (PenCLAHRC), University of Exeter, St Luke’s Campus, Heavitree Road, Exeter, EX1 2LU UK; 7 Exeter Clinical Trials Support Network, Royal Devon & Exeter Foundation NHS Trust, Barrack Road, Exeter, EX2 5DW UK; 8 School of Education, University of Roehampton, Roehampton Lane, London, SW15 5PJ UK; 9 Department of Health Sciences, University of York, Area 2 ATB/152 Seebohm Rowntree Building, Heslington, York, YO10 5DD UK; 10 Torbay Depression and Anxiety Service, 266 Torquay Road, Paignton, TQ3 2EZ UK; 11 RSA (Royal Society for the encouragement of Arts, Manufactures and Commerce), 8 John Adam Street, London, WC2N 6EZ UK; 12 Peninsula Cerebra Research Unit (PenCRU), University of Exeter Medical School, St. Luke’s Campus, Heavitree Road, Exeter, EX1 2LU UK

**Keywords:** Parenting, Early intervention, Group psychotherapy, Child behavioural and emotional problems, Randomised controlled trial

## Abstract

**Background:**

There is a need to build the evidence base of early interventions promoting children’s health and development in the UK. Malachi Specialist Family Support Services (‘Malachi’) is a voluntary sector organisation based in the UK that delivers a therapeutic parenting group programme called *Inspiring Futures* to parents of children identified as having behavioural and emotional difficulties. The programme comprises two parts, delivered sequentially: (1) a group-based programme for all parents for 10–12 weeks, and (2) one-to-one sessions with selected parents from the group-based element for up to 12 weeks.

**Methods/design:**

A randomised controlled trial will be conducted to evaluate Malachi’s *Inspiring Futures* parenting programme. Participants will be allocated to one of two possible arms, with follow-up measures at 16 weeks (post-parent group programme) and at 32 weeks (post-one-to-one sessions with selected parents). The sample size is 248 participants with a randomisation allocation ratio of 1:1. The intervention arm will be offered the *Inspiring Futures* programme. The control group will receive services as usual. The aim is to determine the effectiveness of the *Inspiring Futures* programme on the primary outcome of behavioural and emotional difficulties of primary school children identified as having behavioural or emotional difficulties.

**Discussion:**

This study will further enhance the evidence for early intervention parenting programmes for child behavioural and emotional problems in the UK.

**Trial registration:**

Current Controlled Trials ISRCTN32083735. Retrospectively registered 28 October 2014.

## Background

Longitudinal research indicates that serious anti-social behaviour can be predicted in childhood [1] and parenting, particularly poor parental monitoring, psychological control and negative aspects of support such as rejection and hostility, has been linked to delinquency [2]. The UK scored poorest for child behaviours and risk-taking as well as family and peer relationships and child subjective well-being in a UNICEF comparative analysis of 21 developed countries [3], indicating a need for the implementation of evidence-based early intervention programmes in the UK. The British Child and Adolescent Mental Health Surveys in 1999 and 2004 found that 1 in 10 children and young people between the age of 5 to 15 years had a diagnosable mental disorder [4]. The latest data from the UK Household Longitudinal Survey (Understanding Society) from 2011 to 2012 suggest that 1 in 8 children aged 10 to 15 reported symptoms of mental ill-health [5]. Left unchecked, childhood behavioural difficulties elevate children’s risk for poor outcomes across multiple domains, including academic achievement, health, social relationships and offending [6–12].

Systematic reviews of group-based parenting programmes indicate positive intervention outcomes across measures of child behaviour (for children aged 3–12 years) as well as parental practices and psychological morbidity [13, 14]. The latter review included a meta-analysis that showed a standardised mean difference (SMD) of − 0.53 for parent-report measures of child conduct problems post-treatment. Analyses of the effectiveness of programme components within a meta-analytical review of 77 evaluations of parenting programmes designed to enhance the behaviour and adjustment of 0–7 year-old children reported that programmes that included the following components were predictive of significantly larger effects (*p* < 0.05) on child externalising behaviour: positive interactions with child; responsiveness, sensitivity and nurturing; emotional communication; time out; problem solving; curriculum or manual; modeling; practice with own child; and ancillary services [15].

The majority of these components are central to the Malachi Specialist Family Support Services (‘Malachi’) *Inspiring Futures* programme [16], such as creating positive interactions with the child, responding sensitively to the child’s needs, and emotional communication. Malachi is a UK-based voluntary sector organisation that delivers a therapeutic parenting programme to parents of children with behavioural and emotional difficulties. While teaching parenting skills, such as praise and effective communication, is part of the programme, it also has counselling components, such as developing parents’ awareness of their own early adverse experience and how these influence their current coping strategies, parenting and empathy.

**Fig. 1:**
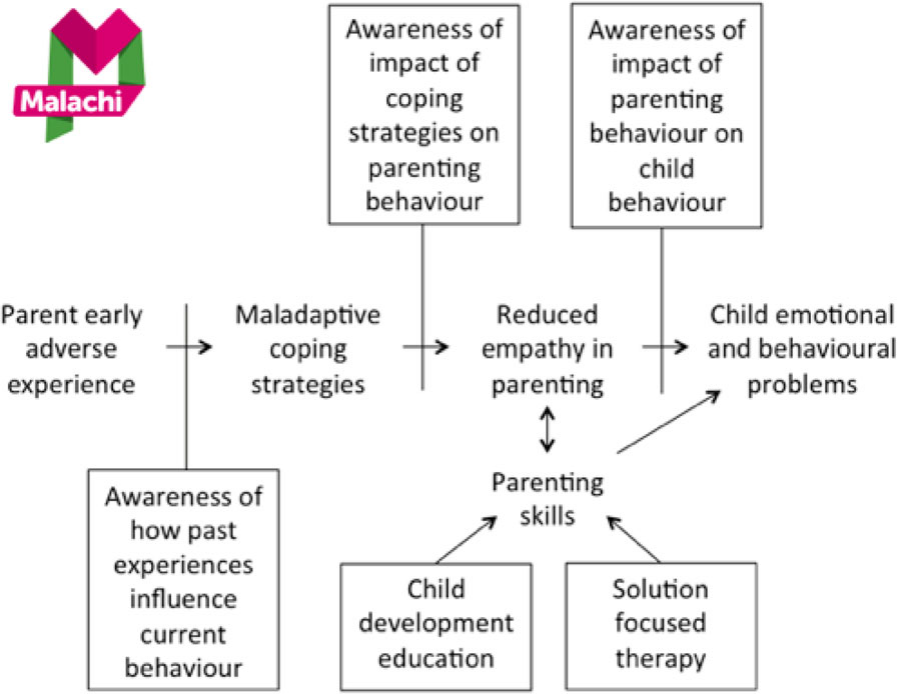
Logic model for Malachi’s *Inspiring Futures* parenting programme

The programme logic model is shown in Fig. 1. Parents with adverse early experiences are more likely to use maladaptive coping strategies and show reduced empathy in parenting, which in turn contribute to their children displaying emotional and behavioural problems [17–19]. *Inspiring Futures* aims to break these causal links in part by increasing parent awareness of how (i) past experiences influence current behaviour, (ii) maladaptive coping strategies affect parenting behaviour and (iii) parenting behaviour affects child behaviour; this approach has arguably been used more with younger children to date [20, 21]. The programme also uses child development education (a component of many proven group-based parenting programmes) and solution-focused therapy [22] to help parents develop better parenting skills.

A previous study of *Inspiring Futures* explored its perceived impact using Interpretive Phenomenological Analysis with six parents who had received the programme (including the one-to-one sessions) [23]. Parents felt supported by Malachi workers and reported that their parenting skills and family relationships had improved.

## Methods

### Objectives

The objectives of the trial are to:

1. Estimate the effectiveness of the *Inspiring Futures* programme on the primary outcome, namely the behaviour and emotional well-being of children with parent-reported emotional and behavioural difficulties.
2. Estimate the impact of the *Inspiring Futures* programme on secondary outcomes, namely (i) parent coping strategies, (ii) empathy in parenting, and (iii) parenting skills (these are all potential mediators).
3. Describe the extent to which the *Inspiring Futures* programme is implemented as intended (i.e., with fidelity to the programme design).

It is hypothesised that, when compared with children whose parent(s) have *not* been offered the programme (the control arm), children whose parent(s) have been offered Malachi’s *Inspiring Futures* programme (the intervention arm) will demonstrate fewer emotional and behavioural difficulties (as reported by parents) (i) at 16 weeks (post-parent group) and (ii) at 32 weeks (post-one-to-one parent sessions, which apply to a selection of parents from the parent group – see ‘Intervention’ below). The primary outcome is at the 32-week follow-up. It is further hypothesised that parents in the intervention arm will demonstrate less maladaptive coping strategies (i.e. their own intervention outcome), greater empathy in parenting, and better parenting skills compared to parents in the control arm (as reported by parents).

### Design

**Fig. 2:**
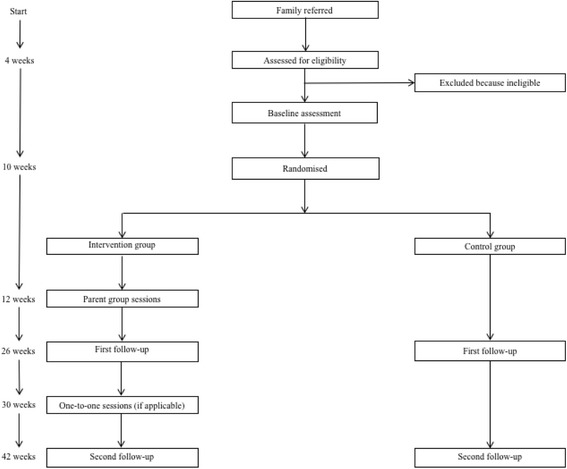
Trial flow chart

A two-arm, randomised controlled, parallel group, superiority trial will be conducted to evaluate the effectiveness of Malachi’s *Inspiring Futures* parenting programme in improving behavioural and emotional outcomes in primary school children with elevated psychosocial difficulties. Two hundred fourty eight participants will be randomised with an allocation ratio of 1:1. Parents in the intervention arm will be offered the *Inspiring Futures* programme; parents in both trial arms will have access to services as usual (with the exception of other Malachi services). The trial therefore examines the superiority of the programme relative to a comparator that represents what young people and their families normally receive. Assessments will take place at baseline, 16 weeks after randomisation (first follow-up, post- group parent programme) and 32 weeks after randomisation (second follow-up, post- one-to-one sessions with selected parents) – data will be collected from all participants at this second follow-up, regardless of whether they have received the one-to-one sessions. (See Fig. 2 for an overview of the trial flow chart and assessments.)

### Participants

The sample will be drawn from approximately 100 mainstream primary schools and children’s centres across the two trial sites in England: Birmingham (c.80) and Somerset (c.20). The former is a large ethnically diverse city while the latter is a large rural county (the trial involves two large towns in the county). The vast majority of referrals (> 90%) are expected to come from schools rather than children’s centres. The minimum number of eligible referred and baselined parents per school needs to be 8 to ensure the minimum viable intervention group size (4) is reached following randomisation. If fewer than 8 parents are recruited from a given school, there is an option to include eligible parents from a nearby school.

Participants will be the parents of children aged 6-11 years at the point of referral (in school years 2 to 6) who are referred by school/centre staff to Malachi’s *Inspiring Futures* Programme. The programme can facilitate the inclusion of partners who are parents of the same child; however, only the main caregiver (as identified by the parents) will report on study outcome measures. Children will be reported to display psychosocial difficulties in the home context, identified by a parent-report ‘borderline’ (or above) Total Difficulties score (i.e. ≥14) on the Strengths and Difficulties Questionnaire (SDQ) [24]. The following exclusion criteria will apply: (i) a parental mental health issue, substance abuse issue or significant self-esteem and/or confidence issue *that would seriously affect their ability to participate in therapeutic group sessions*; (ii) a family situation that does not allow the parent to fully engage in the process (e.g. they do not have enough access to their children to effect change); (iii) other reasons that prevent the parent from participating in the group (not proficient in English, physical health issues, childcare obligations, work commitments); and (iv) parent is already receiving other therapeutic support (e.g. psychotherapy, psychiatry).

### Control arm

Children and parents assigned to the control arm will receive services as usual, because the aim of the trial is to determine whether *Inspiring Futures* provides added value. Malachi state that the support on offer to the kind of children and families targeted by the intervention varies depending on school setting and area, although there may be other organisations that cover the whole city/area. The offer is likely to include support from a school pastoral team or voluntary organisations, and there are also parenting programmes run by local children’s centres. However, whereas other programmes tend to focus on the practical aspects of parenting, Malachi focus on parents’ own experience of being parented and subsequent attachment styles. Any services that parents or children do receive, including other parenting programmes, will be captured in a service use questionnaire (see below). In addition, referrers will be signposted to a standard universal children’s services directory available in Birmingham and Somerset, which may be used to refer children to other services.

### Intervention arm

The *Inspiring Futures* programme comprises two parts, delivered sequentially: (i) a group-based element for all parents, and (ii) one-to-one sessions with selected parents from the group-based element. The Malachi group facilitators, who come from various backgrounds and do not have to be parents themselves, are trained to or working towards at least Level 3 in counselling (or equivalent) and attend an intensive training session lasting 3–4 days. During this training, they first participate in *Inspiring Futures* themselves to ensure that they are familiar and comfortable with the areas covered. They are then trained in the delivery of the programme. Additionally, group facilitators receive monthly supervision by a qualified counsellor throughout the course.

### Group-based element

Parents are invited to attend 10 to 12 (90-min) weekly group sessions (between 4 and 10 parents) at their child’s school facilitated by a pair of Malachi group facilitators. The group sessions run during a school term and the number of sessions depends on the length of the term, but the same content is covered across all groups. The content focuses on helping parents to: understand their children’s needs and the consequences of not meeting them [via child development education]; develop empathy by understanding how children’s thoughts and feelings inform their behaviour; reflect on how their (parents’) parenting behaviours and coping mechanisms affect their ability to meet their children’s needs, and learn how their own experience of being parented (especially adverse experiences) shaped these [using elements of psychodynamic and transactional therapy]; identify and choose to avoid unhealthy default parenting behaviours or maladaptive coping mechanisms (such as the misuse of alcohol or substances, or reckless or self-harming behaviours) and instead pursue more healthy approaches [using solution-focused therapy]; consider how their support network affects their parenting and their children positively or negatively and, if necessary, make changes; and appreciate how conflicts occur and get resolved, their (parents’) default role in conflict and how they can better resolve conflict. Sessions 1 to 7 cover key concepts and theories, sessions 8 to 11 entail participants discussing how to apply their learning in everyday scenarios (these can be consolidated into two sessions), and session 12 includes a recap and discussion of key learning points and the presentation of certificates to parents.

Each session follows the same structure, although there is scope within this to tailor content according to the experiences and engagement of participants. First, Malachi group facilitators attempt to raise the awareness of child development by introducing and explaining psychological theories, such as attachment theory (content, delivery instructions and accompanying handouts are provided to the Malachi facilitators in the programme handbook). Second, perspective taking (i.e. understanding the difficulty of implementing the theory that has been delivered) is promoted in order to alleviate any feelings of guilt that parents might experience as a result of the learning that has taken place (a perspective taking statement is provided for each session in the programme handbook e.g. *‘Not meeting the needs of a child shows that there are things that get in the way of us doing so. Hopefully we can identify what gets in the way for each of us so we can start to do something about our own personal obstacles’*). Third, parents are encouraged to think about how the topic they have just learned about impacts on their life. This is usually the longest part of the session; it can include storytelling, self-disclosure, practical activities and/or reflecting on personal experiences quietly. The Malachi facilitators are trained to facilitate this part of the session by self-disclosing, in other words sharing relevant examples from their own life. The purpose of self-disclosure, which is common in some but by no means all therapeutic approaches, is to normalise experience and encouraging sharing. The topics used in self-disclosure are discussed with the Malachi facilitator’s supervisor prior to the session to assess the appropriateness of the disclosure. (Disclosure is also covered in initial training.) Fourth, most sessions conclude with a ‘homework’ task that is focused around implementing change over the next week; parents are asked to reflect on how well they think they managed the task at the following session (using Likert-type scales tailored to each task). These personal reflections can be shared verbally with the group, or submitted in confidence in a sealed envelope to the Malachi workers if preferred.

### One-to-one sessions

The one-to-one component of *Inspiring Futures* is delivered to a selection of parents who attended the group-based element. This takes place during the term following the group programme delivery in one or more locations agreed between the respective faciitator and parent. Within 7 days of the last group session each Malachi facilitator pair meets to discuss potential referrals into the one-to-one support. The following factors are considered by Malachi when determining eligibility for this further support: (i) parent difficulty in applying the ‘homework’ tasks (as determined by the aforementioned Likert-type scales); (ii) examples of the parent having difficulty connecting past experiences to the present day; (iii) examples of parent difficulty in recognising the impact of their parenting behaviour on the child; and (iv) self-disclosure of an issue that suggests the parent has significant unresolved emotional issues. The additional support initially runs for 6 weeks (with one 60-min session per week) and recaps sessions 2 to 7 of the *Inspiring Futures* programme; no new content is introduced but the one-to-one delivery is intended to allow for deeper levels of discussion with the parent. At week 6 a review takes place with the Malachi worker and the parent to decide whether further input is needed for the remainder of the school term (i.e. a further 4–6 weeks depending on term length); support does not extend beyond the end of the school term. Where possible, a Malachi worker from the pair who led the relevant group element conducts the one-to-one sessions; however, capacity restraints may lead to a Malachi worker who is new to the parent (but trained in delivering the intervention) taking on the role. The worker is consistent across all one-to-one sessions for any given parent.

### Participant timeline

A schematic diagram of the participant timeline can be found in Fig. 2. Once a child is referred to Malachi’s service by a member of school staff (e.g. a teacher or SENCO [Special Educational Needs Coordinator]), eligibility will be assessed using the parent SDQ total difficulties score at a parent information session hosted by Malachi at the child’s school. For those who are eligible, data on additional baseline measures will be collected by a researcher in a home visit. Randomisation will take place 10 weeks after referral. There are two follow-up points to assess changes in outcomes. The first, 16 weeks after randomisation, is equivalent to the end of the parent group part of the programme. The second, 32 weeks after randomisation, is equivalent to the end of the one-to-one part of the programme. The two parts of the programme are delivered over consecutive school terms. The time between processing a referral and the second follow-up is about 9 months.

### Outcome measures

The SDQ Total Difficulties score is the primary outcome; all other outcomes described below are secondary.

#### Strengths and Difficulties Questionnaire (SDQ) [24]

The study will primarily measure changes in levels of children’s behavioural and emotional difficulties from the main parent’s perspective using the SDQ (4–17 years), a 25-item questionnaire comprising 5 subscales (each with 5 items) assessing conduct, hyperactivity, emotional difficulties, peer relations and pro-social behaviour respectively. Each item has three response options (0 = Not true, 1 = Somewhat true, 2 = Certainly true). Scores on each subscale can range from 0 to 10, with higher scores indicating more problems, except for the prosocial subscale where higher scores indicate more prosocial behaviour. The Total Difficulties Score is calculated by summing the 20 items in the first four subscales (range 0 to 40, with higher scores indicating greater problems) and will be used as the primary outcome. The five subscale scores will all be secondary outcomes.

The parent SDQ also has a brief Impact supplement, which starts with a single question about whether the child has difficulties with emotions, concentration, behaviour, or being able to get on with other people (No, Yes – minor difficulties, Yes – definite difficulties, and Yes – severe difficulties). If respondents answer Yes there are four additional questions, focusing respectively on: duration of difficulties (Less than a month, 1–5 months, 6-12 months, Over a year); distress to the child (Not at all, Only a little, Quite a lot, A great deal); interference with the child’s everyday life in terms of home life, friendships, classroom learning and leisure activities respectively (response options as before); and burden to the parent or the family as a whole (response options as before). The parent report impact score is calculated by summing responses to five items, namely (i) whether the difficulties upset or distress the child, and interference with (ii) home life, (iii) friendships, (iv) classroom learning and (v) leisure activities, with the total score ranging from 0 to 10, where higher scores indicate greater impact.

The SDQ has good psychometric properties (e.g. internal consistency is α = 0.80 for parent ratings of the total difficulties scale) for identifying children with behavioural and emotional difficulties in clinical and community populations [25, 26].

#### Eyberg Child Behaviour Inventory (ECBI) [27, 28]

The ECBI is a 36-item parent-rated measure of behaviour problems exhibited by children aged 2 to 16 years. The ECBI has two scales: (1) the Intensity scale (α = 0.94) and (2) the Problem scale (α = 0.93) [27]. For the Intensity scale, parents indicate the frequency of each of the 36 behaviours on a 7-point scale (1 = Never to 7 = Always). The possible range for this subscale is 36 to 252 where higher scores indicate a greater frequency of behaviour problems. The Problem scale assesses whether parents consider the child’s behaviour to be a problem for themselves (Yes / No). The range for this subscale is 0–36, where higher scores indicate that behaviours are more problematic. There is no total composite score. The scale shows good validity for internalising and externalising behaviour problems when compared with the Child Behaviour Checklist (CBCL) [29–31].

#### Ways of Coping Questionnaire (WCQ) [32]

The WCQ is a 66-item self-report measure of coping skills with 50 ‘critical’ items forming an 8-factor structure with acceptable to good internal consistency: confrontive coping (α = 0.70: 6 items); distancing (α = 0.61: 6 items); self-controlling (α = 0.70: 7 items); seeking social support (α = 0.76: 6 items); accepting responsibility (α = 0.66: 4 items); escape-avoidance (α = 0.72: 8 items); planful problem solving (α = 0.68: 6 items); and positive reappraisal (α = 0.79: 7 items). Only the 50 ‘critical’ items will be administered in the present study, as permitted by the developer. The respondent is asked to think of the most stressful situation that they have experienced in the last week and rate how they coped with it. Possible responses for each item range from 0 (“Does not apply or not used”) to 3 (“Used a great deal”). Each subscale is scored by summing the score for each item in the subscale to get a total subscale score. The numbers of items per subscale differ, resulting in different ranges of possible scores across the subscales as follows: confrontive coping: 0 to 18; distancing: 0 to 18; self-controlling: 0 to 21; seeking social support: 0 to 18; accepting responsibility: 0 to 12; escape-avoidance: 0 to 24; planful problem solving: 0 to 18; and positive reappraisal: 0 to 21. There is no overall total score for this measure. Relative scores can be calculated by dividing the average score for each subscale by the sum of the averages for all 8 subscales.

#### Empathy subscale of Adult-Adolescent Parenting Inventory (AAPI-2) [33]

The AAPI-2 has been used in previous evaluations of parenting programmes [34]. A 10-item subscale of the AAPI-2 measuring ‘empathy towards children’s needs’ will be used in the present study. The items assess the understanding and recognition of children’s feelings and needs. Parents are asked to rate their agreement with a statement on a 5-point scale (1 = Strongly Agree, 2 = Agree, 3 = Uncertain, 4 = Disagree, 5 = Strongly Disagree). The range for the total score for this item is 10 to 50, with higher scores indicating greater parental empathy. There are two versions of the inventory (Form A and Form B); for consistency across the three time points, only the empathy subscale of Form A (α = 0.84) will be used in the present study.

#### Alabama Parenting Questionnaire (APQ) [35]

The APQ is a measure of parenting practices. Three of the five subscales will be included in the study: positive involvement with children (10 items, range of possible values for subscale 10 to 50); use of positive discipline techniques (6 items, range of subscale 6 to 30), and consistency in the use of such discipline (6 items, range of subscale from 6 to 30). Each item refers to a parenting practice and respondents are required to indicate how often they typically use each of these practices on a 5-item scale (1 = Never to 5 = Always). The APQ has adequate psychometric properties; the included subscales demonstrate acceptable to good internal consistency (α = 0.67–0.80) [36].

### Other measures

#### Family Demographics Questionnaire (FDQ)

The study will use a short questionnaire to gather basic demographic information about the child and their family. It is adapted from one used in the trial of another parenting intervention [37] and includes variables such as date of birth, age, gender, ethnicity, SEN status, education, members of household, relationship quality, family health and financial situation. The data will be used to describe the sample, examine the extent to which demographic characteristics are balanced between trial arms and carry out attrition analyses (i.e. the extent to which participants who drop out from the intervention and control arms are different on variables such as gender, ethnicity, family type, deprivation).

#### Family Service Use Questionnaire (FSUQ)

The study will use a short questionnaire based on the Client Service Receipt Inventory (CSRI). The CSRI (and versions of it) has been used in over 100 studies since it was first developed in the mid-1980s [38, 39]. The FSUQ will record the receipt of targeted school services and additional services, detailing the typical length and number of contacts, and also whether any of the services received were in relation to the child’s behaviour. It will be used to assess what other services participants in the trial receive and in particular what participants in the control arm receive, as this will help to explain the results (e.g., if there is no impact, it could be because control arm participants received significant extra support from other sources).

### Intervention fidelity

In order to promote fidelity, Malachi provides facilitators with all programme materials for delivery (e.g. manual, exercise materials, and printed posters with agendas and course content). It also provides fidelity instructions in the 3–4 day trainings for facilitators and has a 12-month refresher where the programme content and fidelity tools are reviewed during a 1-day training programme. Fidelity monitoring tools have been developed by Malachi in order to monitor and further promote the high-quality delivery of *Inspiring Futures*, including dose, quality, adherence to the core components of the programme and level of parent engagement. The tools include an attendance register, a self-report checklist for Malachi facilitators and a parent feedback form. Malachi will also use an adapted version of the Parent Programme Implementation Checklist (PPIC), an observational tool which provides a global measure of adherence to core components, the quality of delivery and parent responsiveness [40].

### Data collection

Malachi will obtain the baseline SDQ data from the child’s main parent during an information session at the school (or in a few cases over the phone if the parent is unable to attend the information session). The child’s main parent will provide consent to Malachi for their details to be passed to the Trial Coordinator at the Dartington Social Research Unit (DSRU). An appointment will be made for an independent data collector to visit the family home or child’s school to obtain written informed consent from the main parent to take part in the study. Data collectors will be trained to administer informed consent documents and measures and will complete Level 1 safeguarding training via the NSPCC online course. Data collectors will be supervised by the Trial Manager at the DSRU. The remaining baseline assessments will be collected at that visit (usually within 2 weeks after baseline SDQ), and all follow-up data are collected during home visits. Data will be collected via self-report paper-based questionnaires completed by the main parent. Data collectors will provide assistance to low literacy parents for completing the questionnaires if necessary. Translators will accompany data collectors on visits where parents or children require measures to be administered in languages other than English. Implementation fidelity data will be collected by supervisors at Malachi, and shared with the research team, in line with protocols laid out in the staff handbook. Sessions 4 and 8 in every course will be video-recorded by Malachi (with participants’ consent) and coded using the PPIC by two people working independently (one DSRU, one Malachi) who will then agree a single consistent version.

### Sample size

We aim to recruit 248 participants. This sample will allow us to detect a between-group effect size (ES) of d = 0.40 with 80% power at the 5% level of significance, with allowance for up to 20% loss-to-follow-up.

### Recruitment and retention

Malachi are experienced in working with schools to identify suitable participants for *Inspiring Futures* and will be responsible for recruiting a sufficient number of referrals in order to reach the required sample size. Referrals will be made by a member of school staff (e.g. a teacher or SENCO) who knows the child well and has concerns about the child’s behaviour or emotional well-being. Malachi will screen each referral during an informal information session, which will be held at the school (see ‘Data collection’ above).

Several strategies to minimise attrition will be put in place. The Trial Coordinator will keep regularly updated records in a database to track all study participants. Each participant will be given change of address cards to notify the research team of new contact details and phoned up to three times during the trial to confirm that their contact details are correct. A regular newsletter on the progress of the trial will be distributed to participants, and all families will be offered a £10 gift voucher for each of the three home data collection appointments. Data will be collected from all participants who can be contacted and who consent to participate in data collection, regardless of their level of participation in the intervention.

### Randomisation

An online central computer-randomisation service will be provided by an independent trials unit (Exeter CTU) and will conceal the allocation sequence until assignment to group. The randomisation process will require the Trial Coordinator to log in to a password-protected website and enter the relevant data of each newly recruited participant. The first 25% of the total number of recruits at each site (Birmingham and Somerset) will be allocated by simple randomisation with an allocation ratio of 1:1, and then minimisation will be used to minimise the imbalance between the intervention and control arms in terms of age (< 10 or ≥10 years) and gender. The approach will be dynamic in that each case will be randomised as soon as baseline measures have been completed.

### Blinding

Following randomisation, the Trial Coordinator will notify Malachi by secure electronic communication, and the child’s family and the referrer by standard letter, about the resulting group allocation. The participating family will therefore *not* be blind to allocation. Participants will be aware of the group allocation but will be instructed not to reveal this to the data collector at each follow-up assessment. Data collectors will be blind to group allocation, and will report if they inadvertently become unblinded by participants during a data collection visit. In cases where this occurs at the first follow-up, a different data collector will be assigned to visit the family at the second follow-up where possible. Rates of unblinding of data collectors will be reported with the results of the trial. The FSUQ, which is only administered at the final data collection point (second follow-up), does ask participants – in the last question – to indicate whether or not they received the *Inspiring Futures* programme in whole or in part. The purpose of asking the question is to determine if there is treatment contamination – in other words, if any parents in the control group received *Inspiring Futures*. As this is a self-completion questionnaire, this will not necessarily unmask participants to the data collectors. If it does, however, it is the last question in the final data collection point of the study, and so could not be considered to bias the outcome data. Further, since the outcome data will be collected using self-completion questionnaires rather than through observation or interview (unless a participant requests that the data collector administers the questionnaires in interview style), it is considered unlikely that unblinding data collectors at any point in the study will bias the outcome data. Nevertheless, at the two data collection points where it is relevant (first and second follow-ups) the data collector will be asked to report (i) whether the participant indicated which arm of the trial they are in, and, if so, (ii) which arm it is. The Trial Coordinator will keep and manage the randomisation allocation list in a password-protected document; data collectors will have no access to this list. The statisticians will remain blind to the group allocation.

### Statistical methods

Baseline and demographic characteristics will be summarised using means and standard deviations (or medians and interquartile ranges) for continuous variables and percentage for categorical variables. The comparison of the trial arms will use an intention-to-treat framework with participants analysed according to the trial arm they were randomised to, regardless of whether or not they received the intervention. The primary outcome is the SDQ Total Difficulties score at 32 weeks follow-up. The secondary outcomes are: SDQ Total Difficulties score at 16-weeks follow-up; whether the child scored above the clinically relevant cut-point on the SDQ Total Difficulties scale (i.e. ≥14) (16 and 32 weeks follow-up); the SDQ subscale scores (16 and 32 weeks follow-up); the SDQ Impact Supplement score (16 and 32 weeks follow-up); the ECBI frequency and intensity scales (16 and 32 weeks follow-up); the WCQ subscale scores (16 and 32 weeks follow-up); the AAPI-2 empathy scale (16 and 32 weeks follow-up); and the APQ selected subscale scores (16 and 32 weeks follow-up). The trial arms will be compared in crude (unadjusted) analyses presenting the mean difference for continuous outcomes and odds ratio for binary outcomes. Linear mixed effects models (for continuous outcomes and the binary outcome) will be used with group as a random effect in the intervention arm [41]. Adjustment will be made in these comparisons for the stratification factors (age, gender and trial site), ethnicity, socio-economic status, special educational needs, parent education, parent marital status and the baseline score on the outcome being analysed. The adjusted and imputed (see below) analysis will be considered primary. Subject to having sufficient numbers in the sub-groups, tests of interaction will be used to examine whether the effect of the intervention differs across categories based on age (< 10 versus ≥10 years), gender, ethnicity and level of difficulties on the SDQ Total Difficulties score at baseline (borderline [14 to 16] vs. abnormal [≥17]); 95% confidence intervals and *p*-values will be reported with these estimates. The primary analyses will be based on analyses of 20 multiply imputed datasets to handle missing data. This means that, unless they withdraw consent completely, all randomised participants will be included in the analysis – even if they drop out (refused or unable to contact). Fidelity to the delivery of the intervention programme will be summarised using descriptive statistics. It will be assessed in terms of the different dimensions measured (adherence, dose, quality and engagement). A secondary analysis will be undertaken to quantify the extent to which the intervention effect on the parent SDQ Total Difficulties score at 32 weeks follow-up (the primary outcome) is determined by participation in the intervention (percentage of sessions attended). This will involve a complier average causal effect analysis (CACE) [42, 43] on the complete case data.

### Dissemination

At the end of the research study one or more papers describing the results will be submitted for publication in peer-reviewed academic journals. These will document the key findings of the study in relation to the study objectives. Results will be reported at a group level, meaning that results showing the progress of each individual child/parent in terms of outcomes and other factors will not be provided, whether to the parent, the individual child concerned or anyone else. A summary of the findings will be included in a report that will be made publicly accessible, and a layperson’s summary of the findings will be made available to Malachi and shared with study participants.

### Project timetable and milestones

The main milestones are as follows. Ethical approval for the trial was received in October 2014. The trial was registered on 28th October 2014 (ISRCTN32083735). Recruitment and randomisation of participants for the trial began in October 2014 and was completed in September 2016. Data collection also began in October 2014 and is ongoing at the point of article submission (it will be completed in June 2017). The analyses will be conducted in July–October 2017. The expected date of completion is January 2018.

## Discussion

Programmes that have been developed and tested in the US currently dominate the evidence base on what works in early intervention. The UK is home to many innovative programmes, such as Malachi’s *Inspiring Futures* parenting programme, but few of these have undergone the level of robust evaluation necessary to determine their impact on children’s outcomes. This RCT will be instrumental in building the UK evidence base for early intervention parenting programmes.

Funding was obtained as part of The Big Lottery Fund’s Realising Ambition programme, which involves a £25 m investment over 5 years in 25 interventions that are designed to intervene early in order to divert children and young people aged 8–14 away from pathways into crime, thereby giving them a better chance to realise their ambitions. The design, management, data collection and statistical analysis and dissemination in the trial are fully independent of the funder. The trial management and statistical analysis are fully independent of Malachi, and DSRU will have ultimate authority over the publications submitted to peer-reviewed journals.

## Abbreviations

AAPI-2: Adult-Adolescent Parenting Inventory
APQ: Alabama Parenting Questionnaire
CACE: Complier average causal effect
DSRU: Dartington Social Research Unit
ECBI: Eyberg Child Behavior Inventory
Exeter CTU: Exeter Clinical Trials Unit
FDQ: Family Demographics Questionnaire
FSUQ: Family Service Use Questionnaire
PPIC: Parent Programme Implementation Checklist
SDQ: Strengths and Difficulties Questionnaire
WCQ: Ways of Coping Questionnaire

## References

[1] Farrington DP, Welsh BC (2007). Saving children from a life of crime: early risk factors and effective interventions.

[2] Hoeve M, Dubas JS, Eichelsheim VI, van der Laan PH, Smeenk W, Gerris JR (2009). The relationship between parenting and delinquency: a meta-analysis. J Abnorm Child Psychol.

[3] Adamson P, Bradshaw J, Hoelscher P, Richardson D (2007). Child poverty in perspective: an overview of child well-being in rich countries.

[4] Green H, McGinnity A, Meltzer H, Ford T, Goodman R (2005). Mental health of children and young people in great Britain, 2004.

[5] Beardsmore R. Measuring National Well-Being: insights into Children’s mental health and well-being. Office for National Statistics. 2015;

[6] Nagin DS, Tremblay R (1999). Trajectories of physical aggression, opposition, and hyperactivity on the path to physically violent and non-violent juvenile delinquency. Child Dev.

[7] Bailey JA, Hill KG, Oesterle S, Hawkins JD (2009). Parenting practices and problem behavior across three generations: monitoring, harsh discipline, and drug use in the intergenerational transmission of externalizing behavior. Dev Psychol.

[8] Calkins SD, Keane SP (2009). Developmental origins of early antisocial behavior. Dev Psychopathol.

[9] Breslau J, Lane M, Sampson N, Kessler RC (2008). Mental disorders and subsequent educational attainment in a US national sample. J Psychiatr Res.

[10] Patel V, Flisher AJ, Hetrick S, McGorry P. Mental health of young people: a global public-health challenge. Lancet 2007;369:1302-1313. 10.1016/S0140-6736(07)60368-7 17434406

[11] Fletcher JM (2010). Adolescent depression and educational attainment: results using sibling fixed effects. Health Econ.

[12] Roza SJ, Hofstra MB, van der Ende J, Verhulst FC (2003). Stable prediction of mood and anxiety disorders based on behavioral and emotional problems in childhood: a 14-year follow-up during childhood, adolescence, and young adulthood. Am J Psychiatr.

[13] Barlow J, Smailagic N, Bennett C, Huband N, Jones H, Coren E. Individual and group based parenting programmes for improving psychosocial outcomes for teenage parents and their children (review). Chichester: Wiley; 2012. 10.1002/14651858.CD002964.pub2 PMC4164375 21412881

[14] Furlong M, McGilloway S, Bywater T, Hutchings J, Smith SM, Donnelly M. Behavioural and cognitive-behavioural group based parenting programmes for early-onset conduct problems in children aged 3 to 12 years (review): The Cochrane Collaboration: Wiley; 2012. 10.1002/14651858.CD008225.pub2 PMC12935172 22336837

[15] Kaminski JW, Valle LA, Filene JH, Boyle CL (2008). A meta-analytic review of components associated with parent training program effectiveness. J Abnorm Child Psychol.

[16] Malachi Specialist Family Support Services (2014) Inspiring Futures Parenting Programme. Birmingham: Malachi Specialist Family Support Services CLC.

[17] Felitti VJ, Anda RF, Nordenberg D, Williamson DF, Spitz AM, Edwards V, Koss MP, Marks JS (1998). Relationship of childhood abuse and household dysfunction to many of the leading causes of death in adults: the adverse childhood experiences (ACE) study. Am J Prev Med.

[18] Murphy A, Steele M, Dube SR, Bate J, Bonuck K, Meissner P (2014). Adverse childhood experiences (ACEs) questionnaire and adult attachment interview (AAI): implications for parent child relationships. Child Abuse Negl.

[19] Luyten P, Nijssens L, Fonagy P, Mayes LC (2017). Parental reflective functioning: theory, research, and clinical applications. The Psychoanalytic Study of the Child.

[20] Lieberman AF, Van Horn P (2008). Psychotherapy with infants and young children: repairing the effects of stress and trauma on early attachment.

[21] Barlow J, Bennett C, Midgley N, Larkin S, Yinghui W. Parent-infant psychotherapy for improving parental and infant mental health. Cochrane Database Syst Rev. 2015 Jan 8;1:CD010534. 10.1002/14651858.CD010534.pub2. 10.1002/14651858.CD010534.pub2 PMC8685508 25569177

[22] De Shazer S, Dolan Y, Korman H, Trepper T, McCollum E, Kim BI (2007). More than miracles: the state of the art of solution-focused brief therapy.

[23] Hickman G. Parents’ understanding of the impact of the Malachi Community trust intervention on their parenting behaviour: an interpretive phenomenological analysis*.* 2007; Masters dissertation.

[24] Goodman R (1997). The strengths and difficulties questionnaire: a research note. J Child Psychol Psychiatry.

[25] Goodman R (2001). Psychometric properties of the strengths and difficulties questionnaire. Journal of the American Academy of Child & Adolescent Psychiatry.

[26] Stone LL, Otten R, Engels RC, Vermulst AA, Janssens JM (2010). Psychometric properties of the parent and teacher versions of the strengths and difficulties questionnaire for 4- to 12-year-olds: a review. Clin Child Fam Psychol Rev.

[27] Eyberg S, Ross AW (1978). Assessment of child behaviour problems: the validation of a new inventory. Journal of. Clinical Child Psychology and Psychiatry.

[28] Eyberg SM (1980). Eyberg child behaviour inventory. Journal of Clinical Child Psychology..

[29] Burns GL, Patterson DR (2001). Normative data on the Eyberg child behavior inventory and Sutter-Eyberg student behavior inventory: parent and teacher rating scales of disruptive behavior problems in children and adolescents. Child Family Behav Ther.

[30] Boggs SR, Eyberg S, Reynolds LA (1990). Concurrent validity of the Eyberg child behavior inventory. Journal of Clinical Child Psychology..

[31] Achenbach TM, Edelbrock CS (1983). Manual for the child behavior checklist and revised child behavior profile.

[32] Folkman S, Lazarus RS (1988). Ways of coping questionnaire manual.

[33] Bavolek SJ, Keene RG. Adult-adolescent parenting inventory AAPI-2: administration and development handbook. Park City, UT: Family Development Resources, Inc; 2001.

[34] Marcynyszyn LA, Maher EJ, Corwin TW (2011). Getting with the (evidence-based) program: an evaluation of the incredible years parenting training program in child welfare. Child Youth Serv Rev.

[35] Frick PJ (1991). The Alabama parenting questionnaire.

[36] Shelton KK, Frick PJ, Wootton J (1996). Assessment of parenting practices in families of elementary school-age children. Journal of Clinical Child Psychology.

[37] Hutchings J, Bywater T, Daley D, Gardner F, Whitaker C, Jones K, Eames C, Edwards RT (2007). Parenting intervention in sure start services for children at risk of developing conduct disorder: pragmatic randomized controlled trial. Br Med J.

[38] Beecham J. The Client Service Receipt Inventory (CSRI). Report prepared for the Concerted Action Research Programme, European Commission, Brussels. CEMH Working Paper 053; 1995.

[39] Chisholm D, MRJ K, Knudsen HC, Amaddeo F, Gaite L, van Wijngaarden B, The EPSILON Study Group (2000). Client socio-demographic and service receipt inventory – EU version: development of an instrument for international research. Br J Psychiatry.

[40] Bywater T, Berry V, Tobin K, Gridley N, Blower S. Development and validation of the Parent Programme Implementation Checklist: A Short Observational Tool to Assess Quality of Delivery', Working Paper (contact tracey.bywater@york.ac.uk); 2016.

[41] Flight L, Allison A, Dimairo M, Lee E, Mandefield L, Walters S (2016). Recommendations for the analysis of individually randomised controlled trials with clustering in one arm – a case of continuous outcomes. BMC Med Res Methodol.

[42] Hewitt C, Torgerson D, Miles J (2006). Is there another way to take account of noncompliance in randomized trials?. Can Med Assoc J.

[43] Dunn G, Bentall R (2007). Modelling treatment-effect heterogeneity in randomized controlled trials of complex interventions (psychological treatment). Stat Med.

